# High food insecurity in Latinx families and associated COVID-19 infection in the Greater Bay Area, California

**DOI:** 10.1186/s40795-021-00419-1

**Published:** 2021-06-11

**Authors:** Milagro Escobar, Andrea DeCastro Mendez, Maria Romero Encinas, Sofia Villagomez, Janet M. Wojcicki

**Affiliations:** Division of Pediatric Gastroenterology, Hepatology and Nutrition, University of California, 550 16th Street, 5th Floor, San Francisco, CA 94158-0136 USA

**Keywords:** COVID-19, Latinx, Food insecurity, Children

## Abstract

**Background:**

Food insecurity impacts nearly one-in-four Latinx households in the United States and has been exacerbated by the novel coronavirus or COVID-19 pandemic.

**Methods:**

We examined the impact of COVID-19 on household and child food security in three preexisting, longitudinal, Latinx urban cohorts in the San Francisco Bay Area (*N* = 375 households, 1875 individuals). Households were initially recruited during pregnancy and postpartum at Zuckerberg San Francisco General Hospital (ZSFG) and UCSF Benioff prior to the COVID-19 pandemic. For this COVID-19 sub-study, participants responded to a 15-min telephonic interview. Participants answered 18 questions from the US Food Security Food Module (US HFSSM) and questions on types of food consumption, housing and employment status, and history of COVID-19 infection as per community or hospital-based testing. Food security and insecurity levels were compared with prior year metrics.

**Results:**

We found low levels of household food security in Latinx families (by cohort: 29.2%; 34.2%; 60.0%) and child food security (56.9%, 54.1%, 78.0%) with differences between cohorts explained by self-reported levels of education and employment status. Food security levels were much lower than those reported previously in two cohorts where data had been recorded from prior years. Reported history of COVID-19 infection in households was 4.8% (95% Confidence Interval (CI); 1.5–14.3%); 7.2% (95%CI, 3.6–13.9%) and 3.5% (95%CI, 1.7–7.2%) by cohort and was associated with food insecurity in the two larger cohorts (*p* = 0.03; *p* = 0.01 respectively).

**Conclusions:**

Latinx families in the Bay Area with children are experiencing a sharp rise in food insecurity levels during the COVID-19 epidemic. Food insecurity, similar to other indices of poverty, is associated with increased risk for COVID-19 infection. Comprehensive interventions are needed to address food insecurity in Latinx populations and further studies are needed to better assess independent associations between household food insecurity, poor nutritional health and risk of COVID-19 infection.

**Supplementary Information:**

The online version contains supplementary material available at 10.1186/s40795-021-00419-1.

## Background

Food insecurity is common among Latinx families, the largest ethnic minority group in the United States, and has been further exacerbated by the novel coronavirus pandemic (COVID-19) [1]. Prior to COVID-19, 1 in 9 households faced food insecurity with a higher prevalence in Latinx families [2, 3]. Latinx individuals comprise over 60 million individuals in the United States [4] and are twice as likely as White Americans to face poverty including aspects of food insecurity [5]. Preliminary reports since the beginning of the COVID-19 shelter-in-place lockdowns in the United States (approximately March 2020) suggest that Latinx families have had higher increases in food insecurity compared to Whites (32% versus 18%) with even higher rates in families with children [6].

Higher rates of food insecurity in Latinx communities can be explained by sociodemographic and economic factors specific to these communities. Latinx individuals are more likely to be low-income, recent immigrants, have young children, live in larger households, and reside in urban areas that have been historically prone to food insecurity [7–10]. Furthermore, Latinx adults may have been most impacted by loss of employment due to sectors that were particularly impacted by COVID-19 shutdowns including the hotel industry, food services, healthcare, and manufacturing [11]. Similar to other high-risk groups, Latinx families have relied on government food services since the start of the pandemic [12]. However, the number of Latinx families served is likely underestimated as many Latinx immigrants who contribute to the US economy are not able or resistant to receiving economic relief in context of the COVID-19 pandemic due to lack of legal status. Additional disparities in Latinx communities include crowded living conditions, housing insecurity, education gaps, few family resources, all which create higher risk for COVID-19 infection [13].

We have previously assessed the prevalence of food insecurity in Latinx low-income families residing in six counties of the San Francisco Bay Area finding high rates prior to COVID-19 [14]. Slightly more than half of the households previously surveyed had high food security (58.3%) whereas the remainder experienced marginal food security (8.6%), low food security (28.2%), and very low food security (4.9%). Food insecurity in children has been linked to lower academic performance, anxiety, obesity and diabetes. Adults with food insecurity can have lower scores on mental and physical health assessments higher risk of depression and anxiety, chronic disease, and poorer overall health status. Previous studies have also suggested that food insecurity may increase risk for infectious disease such as HIV in the context of exposure due to poor immunological health [15].

To our knowledge, no study has investigated the impact of COVID-19 on food security in a longitudinal, Latinx sample of participants residing in an urban U.S. context. We examined food security during COVID-19 using three prospective longitudinal studies of Latinx households based in the 6 counties of the San Francisco Bay Area. We evaluated levels of food security and insecurity during COVID-19 compared with previous levels (prior to COVID-19) in these populations as well as the association between COVID-19 infection and food insecurity.

## Methods

### Recruitment and consent

Mothers were recruited from three previous longitudinal birth cohort studies: the Hispanic Eating and Nutrition Study (HEN) (*n* = 201) [16, 17], Latinx, Eating, and Diabetes Study (LEAD) (*n* = 97) [18, 19] and the Telomeres At Birth Study (TAB) (*n* = 424). Participants for both HEN and LEAD were recruited during pregnancy as previously described and TAB participants were recruited post-partum before being discharged from the hospital. All participants from HEN, LEAD and TAB have been followed up over the course of approximately 13, 8, and 1–2 years respectively. These cohorts were initially recruited to evaluate the relationship between prenatal and early postnatal exposures and development of childhood obesity. Inclusion criteria for HEN and LEAD included self-identifying as Latinx and expecting a healthy newborn infant as previously described. Participants for HEN and LEAD were primarily recruited from Zuckerberg San Francisco General Hospital (ZSFG) with a smaller percentage from UCSF Benioff for HEN [17, 19]. These two cohorts consist of families of primarily Spanish-speaking foreign-born adults with less than a high school education and originating primarily from the countries of Central America and Mexico [17, 19]. We initially recruited all race/ethnic backgrounds for TAB but restricted entry criteria to Latinx for the second phase of our study. TAB participants were recruited from UCSF Benioff (70.2%) and ZSFG (29.8%) and represent a broader background of education levels, countries of ancestry and place of birth.

To assess participation in the COVID-19 food insecurity sub-study, participants were contacted by telephone during the months of May to September 2020 using previous contact information. Less than 10% of those that were successfully contacted declined participation, although we were not able to contact a significant percentage of our cohorts due to a limited time period of recruitment. Participants were asked for their language preference (English or Spanish) and were provided information about the COVID-19 sub-study by bilingual research coordinators and the study principal investigator. We obtained verbal consent from those participants who were interested, and participants took part in a 15-minute interview by telephone. Food insecurity was assessed using the US Household Food Security Scale Module (US HFSSM) that we have used annually with HEN and LEAD studies to assess food insecurity [20]. The US HFSSM has been validated for use in Spanish with low -income Spanish speaking adults from Mexico, Central America, Cuba and Puerto Rico [21] and has also been confirmed for face validity through qualitative focus groups with low income pregnant and postpartum Latinx women in the United States [22].

We additionally asked questions about COVID-19 symptoms and testing, housing and employment status and food consumption. Participants were compensated for their time for participation with a $15 gift card to Target or Amazon based on participant preference. The principal investigator of the study (JW) and four research coordinators including (ME, ADC and MR) conducted all interviews. The Committee on Human Research (CHR), the UCSF Institutional Review Board approved all aspects of the study (IRB numbers 11–06334; 16–06163; 16–18535).

### Procedures

Food insecurity was measured using the 18-item US Household Food Security Scale Module. The timeframe of the questions was modified to collect data on food access from when COVID-19 lockdowns first began (March 2020) to the time of interview. The Food Insecurity Module included questions regarding adult and children’s eating habits [20]. Scoring was done automatically by REDCap, with higher scores indicating a higher level of food insecurity. We followed previously established food security categories: high food security or food secure (0 affirmative responses), marginal food security (1–2 affirmative responses), low food security (3–7 affirmative responses), and very low food security (8–18 affirmative responses). Child food security levels were categorized as high food security or food secure (0 affirmative responses), marginal food security (1 affirmative response), low food security (2–4 affirmative response) and very low food security (5–8 affirmative responses). We compared mean ± SD food security scores and categorical markers of food security with prior year assessments of food security in two out of three cohorts (HEN and LEAD). We had no prior assessment of food security in TAB participants. Previous levels of household and child food security collected in the years before the COVID-19 pandemic were compaed with food security levels during the pandemic.

Food assistance information was collected prior to the COVID-19 pandemic as part of our previous longitudinal data collection (self-reported participation in the Special Supplemental Program for Women, Infants and Children’s program (WIC) or participation in the Supplemental Nutrition Assistance Program (SNAP; previously known as food stamps)). Similarly, maternal education level (primary or less than a high school diploma, high school diploma, some college or more) and primary language use (Spanish, English or bilingual) was also collected at study entry for all participants. As part of the COVID-19 sub-study we asked about adult household employment status, number of total household members as well as crowding indices including size of house (number of bathrooms and bedrooms). As the COVID-19 sub-study was a new part of our overall study, we pilot tested the face validity of the questions with the first 10 participants, making subtle initial changes to the questionnaire asking participants to repeat or rephrase their understanding of what the question was asking.

Participants were also asked about COVID-19 symptoms, laboratory-based testing as well as duration of symptoms for all household members since middle of March 2020 until time of interview. Household units were the level of the analysis.

### Statistical analysis

Household and child food security levels and corresponding insecurity were calculated including mean score ± standard deviation (SD) as well as percentages by category. Data was stratified by cohort using Stata 15.0. Levels of household and child food security were assessed for normality using graphical assessments as well as statistical tests to assess normality including Shapiro-Wilk. As there were departures from normality, non-parametric statistical tests were used to assess associations including Wilcoxon rank sum, Kruskal-Wallis and Spearman rank order correlation coefficient for continuous predictors and chi-squared tests for categorical ones. Associations between household and child food insecurity levels (continuous measures and categorical variables) and education level (high school or lower versus more than high school education), language use (Spanish, English or bilingual), COVID-19 testing positivity, and employment status (unemployed or adult household employment) were assessed using non-parametric methods. Multivariable logistic models were used to evaluate independent predictors of household COVID infection in relation to food insecurity in TAB and HEN cohorts adjusting for potential confounders such as unemployment and crowding that were significant at *p* value < 0.05 in bivariate analysis. We did not assess independent predictors of household COVID infection in LEAD using modeling due to the small sample size of that cohort.

## Results

We recruited 200 Latinx families from TAB (250 total recruited including 50 non-Latinx families), 111 from HEN and 64 from LEAD for a total participation of 375 Latinx families. Mean household size was 4.8 ± 1.7 for TAB, 5.3 ± 2.0 for LEAD, and 4.9 ± 1.6 for HEN (Table 1). Total sample size of all household members for all three longitudinal studies combined was 1875. All studies reported a comparable number of children in households with a mean of 2.4 ± 1.1 for TAB, 2.7 ± 1.1 for LEAD and 2.9 ± 1.2 for HEN (Table 1).

**Table 1:** Demographics and Household Specifics by Bay Area Cohort

Cohort Name	Telomeres at Birth (TAB)	Latinx, Eating and Diabetes (LEAD)	Hispanic, Eating and Nutrition (HEN)
	% (N)	% (N)	% (N)
	Mean ± SD	Mean ± SD	Mean ± SD
	***N =*** 200 families	***N*** = 65 families	***N*** = 111 families
Household size			
No. of people	4.8 **±** 1.7	5.3 **±** 2.0	4.9 **±** 1.6
No. of children	2.4 **±** 1.1	2.7 **±** 1.1	2.9 **±** 1.2
Number share bedroom	2.5 **±** 1.0	2.8 **±** 1.3	2.3 **±** 1.1
Number share bathroom	3.3 **±** 1.6	4.5 **±** 1.6	3.8 **±** 1.6
Food Assistance			
SNAP/Food Stamps	Not Collected	47.1%	43.4%
WIC participation	41.4%	15.7%	72.9%
Language Use			
Spanish	38.7%	90.6%	91.1%
English	56.8%	4.7%	8.9%
Bilingual	4.5%	4.7%	–
(English/Spanish)			
Maternal Education			
Highest Education Level			
High School Or less	34.3%	79.4%	75.2%
Employment			
All household adults unemployed	24.1%	35.2%	38.8%

Close to half of HEN and LEAD participants reported receiving SNAP benefits (food stamps) prior to the onset of the COVID-19 pandemic (43.4 and 47.1% respectively). We did not have this information on TAB participants. All of the HEN and LEAD participants self-identified as Latinx with 80.6% of the TAB participants identifying as Latinx (13.8% non-Hispanic Caucasian and 5.3% as Asian including Pacific Islander). We only included those TAB participants in this COVID-19 sub-study who identified as Latinx (*n* = 200).

A high percentage of HEN and LEAD participants reported using Spanish as a primary language (91.1 and 90.5%) with a much lower percentage reporting Spanish as the primarily language (38.7%) for TAB participants. Other sociodemographic differences between HEN, LEAD and TAB included higher maternal education levels for TAB. Among TAB participants, 34.3% had a high school education or less (65.7% with university or postgraduate qualifications) compared with 79.4% of LEAD reporting high school as the highest qualification (20.6% with university/postgraduate) and 75.2% for HEN (24.8% with university/postgraduate). During the COVID-19 pandemic time frame studied, 24.1% of TAB participants, 35.2% of LEAD and 38.8% of HEN households had no adults who were employed.

### Food security

Two out of three cohorts report low rates of food security including LEAD (29.2%) and HEN (34.2%) compared to higher reports of household food security for TAB participants (60%, Table 2). Similarly, a high percentage of participants in HEN and LEAD report low and very low food security (46.2% for LEAD and 49.5% for HEN) with a much lower 26.5% for TAB. Slightly more than half of participants from HEN and LEAD report child food security. LEAD and HEN reported low levels of child food security (56.9 and 54.1% respectively) which was much higher for TAB (78%) (Table 2).

**Table 2:** Levels of Household, Child Food Security and COVD-19 Infection by Bay Area Cohort

	Telomeres at Birth (TAB)	Latinx, Eating and Diabetes (LEAD)	Hispanic, Eating and Nutrition (HEN)
	% (N)	% (N)	% (N)
	Mean ± SD	Mean ± SD	Mean ± SD
	***N =*** 200	***N =*** 65	***N =*** 111
Levels of Household Food Security			
Food Security Score, Mean **±** SD	1.6 **±** 2.7	3.3 **±** −3.7	3.5 **±** 3.6
High Food Security (Food Secure)	60.0 (120)	29.2 (19)	34.2 (38)
Marginal Food Security	13.5 (27)	24.6 (16)	16.2 (18)
Low Food Security	20.5 (41)	33.9 (22)	31.5 (35)
Very Low Food Security	6.0 (12)	12.3 (8)	18.0 (20)
Levels of Child Food Security			
High Food Security (Food Secure)	78.0 (156)	56.9 (37)	54.1 (60)
Marginal Food Security	8.0 (16)	10.8 (7)	10.8 (12)
Low Food Security	12.0 (24)	26.15 (17)	32.4 (36)
Very Low Food Security	4 (2.0)	6.15 (4)	2.7 (3)
COVID Infection History			
Positive test reported March-Sept 2020	7/200 (3.5%)	3/62 (4.8%)	8/111 (7.2%)
	(95%CI; 1.7–7.2%)	(95%CI; 1.5–14.3%)	(95%CI; 3.6–13.9%)

### Associations between food insecurity and socio-demographics

There were no associations between socio-demographics including maternal education level and employment status with household or child food security in HEN or LEAD cohorts. Higher household crowding factors as indicated by more people sharing a bedroom or bathroom were associated with having less household and child food security in HEN but not LEAD cohorts (Rho = 0.29, *p* < 0.01 for number sharing a bedroom for total food security score and Rho = 0.28, *p <* 0.01 for number sharing a bath; Rho = 0.33, *p <* 0.01 for number sharing a bedroom for child food security score and Rho = 0.27, *p <* 0.01 for number sharing a bathroom).

Sociodemographic variables associated with poverty including not being employed and having a lower education level (high school versus any university or postgraduate training) were associated with increased levels of household and child food insecurity in TAB (*p <* 0.01). Furthermore, crowding metrics (persons/bedroom and persons/bathroom) were also significantly associated with household and child food insecurity in TAB (*p* < 0.01). Additionally, being a preferred/dominant Spanish language speaker was also highly correlated with having less household and child food security in TAB (z = − 6.8 *p <* 0.01 household security and z = − 4.2, *p <* 0.01 for child security) but not in HEN or LEAD.

### Change from food insecurity in prior years

Data from these same cohorts in the last year and prior years suggest that there is much more severe food insecurity with fewer families now being food secure during the COVID-19 pandemic. In LEAD interviews from 1 year ago, 76.9% of families were household food secure and 87.9% had child food security. In the 6 months prior to COVID, we assessed a sub-sample of HEN families (*n* = 38) and 79.0% had household security and 89.2% had child food security. A larger study of HEN participants assessed more than 5 years ago (*n* = 161) found that many of these families had struggled with food security in prior years with 56.6% with household food security and 69.3% with child food security.

### Association with COVID-19 infection

There were 8 households surveyed in HEN that had COVID-19 infections as indicated by self-report positive reverse transcription polymerase chain reaction (RT-PCR) tests out of the 111 total households surveyed that also had level of food security reported (7.2, 95%CI; 3.6–13.9%) (Table 2). Of these, 12.5% reported household food security compared with 34.7% of those households without individuals with COVID-19 positive members (*p* = 0.03). Adjusting for previously described crowding factors including persons per bedroom and bathroom, food insecurity category was not associated with COVID-19 positivity (*p* = 0.60). The percentage of COVID-19 positive households was lower among LEAD, 4.8% (95%CI; 1.5–14.3%); 3 out of 62 households having a COVID-19 infection (Table 2). There was no association between COVID-19 infection and household food insecurity in LEAD. Similarly, the infection rate in TAB household was lower than HEN (7 out of 200 or 3.5% (95%CI; 1.7–7.2%)) (Table 2). However, there was also a trend towards reduced food security among TAB households that were infected (42.9% food secure among infected versus 60.6% of those uninfected and 28.6% had the highest level of food insecurity among infected compared with 5.2% of those uninfected, *p* = 0.02). In a multivariable model of the TAB cohort adjusting for employment status, crowding as indicated by number of household members sharing a bathroom and Spanish language use, increased household food insecurity score trended towards a positive association with household COVID infection (OR 1.36, 95%CI 0.97–1.92; *p* = 0.079).

## Discussion

Racial and ethnic minorities historically have worse outcomes during and after disasters as they already face a range of economic, political, and social barriers [23, 24]. These include but are not limited to racial discrimination, exploitation, limited English-speaking proficiency, and lack of legal status [24].

The overall prevalence of food insecurity in California between 2017 and 2019 was 9.9% having low food security and 3.6% with very low food security [25]. Overall national levels of food insecurity were higher during this time period for Hispanic households (15.6%).

Food insecurity has increased for American children and adults since the start of COVID-19 pandemic with elevations in every state [6]. Data from the Census Pulse Household Survey suggest that overall food insecurity tripled from 9.4 to 29.5% for households with children during COVID-19. Similarly, we report dramatic increases in food insecurity in our low income Latinx cohorts in the San Francisco Bay Area. These population groups already had low rates of food security. Prior to COVID-19, 76.9% of LEAD families reported having food security but during COVID-19 only 38.1% report food security. Similarly, HEN food security declined from 79.6% of adults having food security to 34.2%. Decreases in child food security were also significant with 87.9% secure prior to COVID declining 56.9% in LEAD and 89.2 to 54.1% in HEN. Our results in HEN and LEAD correspond with the high food insecurity rates (only 36% with food security) reported by US adults living < 250% below the federal poverty line during the COVID-19 pandemic by Wolfson and Leung (2020) [26].

Our findings of high food insecurity among three Latinx cohorts in the San Francisco Bay Area suggest rapid growth of food insecurity in urban areas impacted by COVID-19 shutdowns. Latinx individuals are among the most likely to be pushed into poverty as a result of pandemic-related unemployment [6, 27]. A significant percentage of our participants did not have any adult household members with employment at the time of the interview (more than one third of HEN and LEAD households and a quarter of TAB), and unemployment is strongly associated with food insecurity [6, 28]. Additionally, Latinx individuals, such as the individuals in cohorts, who faced food insecurity prior to COVID-19 likely have more severe food insecurity during the pandemic and are particularly at risk for adverse outcomes [26].

Common coping strategies for food insecurity prior to COVID-19 include use of food pantries, borrowing money, going to homes of friends or family for meals, or sending children to relatives [29]. Some, if not all of these tactics to buffer families against food insecurity, are more limited given the recommended physical distancing measures being taken to prevent COVID-19 transmission [30]. Even when food pantry resources are available, Latinx families compared with other groups may face language barriers or concerns about legal status which limit use [31]. Shame can also result in people hiding hunger rather than going to food pantries and further increasing food vulnerability [32].

### Differences among Latinx cohorts

There were large differences between our Latinx cohorts in levels of food insecurity. Part of this can be explained by socio-demographic differences by cohort. HEN and LEAD were recruited primarily at Zuckerberg San Francisco General Hospital, the local safety net county hospital with TAB recruited primarily at UCSF Benioff, a tertiary care academic hospital. The women recruited for HEN and LEAD cohorts had lower levels of eduation at recruitment (a low percentage reported any education beyond high school at recruitment (20.6% for LEAD and 26.1% for HEN) versus a much higher 65.7% for TAB). Low education status is the greatest predictor of unemployment [33] and since the emergence of COVID-19, employment losses are associated with low education levels [34]. Higher rates of household and child food insecurity in HEN and LEAD versus a much lower level in TAB (60% with high food security) parallel the lower education levels and potential earning power in these cohorts [6, 28].

Additionally, over 90% of mothers from the HEN and LEAD were preferred/dominant Spanish-speakers while 65% of the TAB mothers were primary English-speaking. Being fluent in English leads to greater job opportunities [35] also potentially contributing to food insecurity [6, 28]. Our previous studies with HEN and LEAD have also found that the majority of adults in these households are recent immigrants and foreign-born [17–19], potentially placing these families at a greater disadvantage in terms of accessing food and other government resources [36].

There was little difference in reported crowding indices (the number sharing bedrooms and bathrooms) between these three cohorts and although there were only associations between these metrics and food insecurity in TAB and HEN possibly based on differences in sample size (smaller sample size and less power in LEAD). Furthermore, we did not see any differences in food insecurity and education level or employment status for LEAD and HEN possibly because there was little variation in education level with the majority not having any advanced education beyond high school. We did not assess for underemployment common among Latinx adults but HEN and LEAD may have higher levels of underemployment [37]. Meanwhile for TAB, the association between education level and employment status and food insecurity was highly significant. Those with less education had greater food insecurity, possibly due to the heterogeneity in education levels for this cohort.

### Necessary interventions for Latinx families

Our data suggest the urgent need for interventions for Latinx families with children, particularly those who are Spanish-speaking and with low education and employment levels in the context of the COVID-19 crisis. Data from our TAB cohort points to the heterogeneity among Latinx families in the Bay Area and the potential need for targeted interventions in specific communities. Due to urgent need, some safety net clinics in California have had creative responses in terms of bringing food to families. More funding and development of these efforts should be prioritized by state and county legislators [38, 39]. Furthermore, individuals from underrepresented groups tend to not have enough accessible information in their preferred language and mode of communication [40]. For instance, 29% of Latinx in the United States have no English fluency [41]. There can also be distrust of the government and health agencies due to poor history and absence cultural competency [36, 40]. The best implementations for COVID-19 relief will be made through the inclusivity of cultural richness, such as having information in multiple languages, modes of communication, and a diverse workforce to implement these interventions and work with diverse communities.

### Risk for COVID-19 infection with household food insecurity

Previous researchers have noted an increased risk for COVID-19 morbidity and mortality due to low quality nutrition, obesity and associated metabolic disease [42]. While crowding factors and poverty are associated with risk for COVID-19 infection and food insecurity, food insecurity may be independently associated with increased risk for COVID-19 infection through poorer immune health and increased susceptibility to disease. Previous studies, particularly related to risk for HIV infection in the context of HIV exposure suggest that food insecurity may be an independent risk factor for infectious disease [15]. For example, other studies suggest that household food insecurity puts children in a more susceptible position for tuberculosis infection [43]. In multivariable analyses adjusting for crowding, unemployment and Spanish language use in the TAB cohort, food insecurity score trended towards association with household COVID infection (OR 1.25, 95%CI 0.97–1.61). Further studies are needed with larger cohorts to assess the role between food insecurity, poor nutritional status and risk for infectious disease, inlcuding COVID-19.

## Conclusions and limitations

Further population-based studies are needed with larger and heterogeneous cohorts to assess the impacts of COVID-19 in other high-risk population groups, in addition to Latinx. Rural communities and suburban areas may face different food insecurity stressors and as such our findings may not be relevant to rural or suburban areas. Government and non-governmental agencies need to prioritize poverty-alleviating measures including mitigating food insecurity as a means to address the public health demands of COVID-19 infection.

Lastly, while we focused on food insecurity, other studies suggest that the COVID-19 epidemic may have had broad ranging impacts of dietary practices. International studies suggest that the COVID-19 epidemic and the prolonged confinement and “stay at home measures” impacted children’s dietary intake including increased intake of sweetened foods such as desserts [44]. Future studies should evaluate the impact of work from home policies and remote schooling on children’s and family’s dietary intake and nutritional health in addition to overall food security.

## Supplementary Information

**Additional file 1.**

## Data Availability

The data from this study is available on written request to the Principal Investigator (JMW).
