# Supplementary material for: High food insecurity in Latinx families and associated COVID-19 infection in the Greater Bay Area, California
Source: BMC Nutr. 2021 Jun 11;7:23. doi: 10.1186/s40795-021-00419-1 (PMC8192129; doi:10.1186/s40795-021-00419-1)
Supplement: Supplementary file 1 — Additional file 1. [file 40795_2021_419_MOESM1_ESM.pdf]

Covid19

Record ID	______________________
Date	______________________
Study Name	______________________
Participant ID numer/SNO	______________________
Participant Name	______________________
1. Have you or your child had any symptoms that are consistent with Covid-19 (thinking back to March until now)?	◯ Yes Adult<br>◯ Yes Child<br>◯ Yes Both<br>◯ No Neither
For how many days did you have these symptoms?	______________________
For how many days did your child have these symptoms?	______________________
Were these symptoms confirmed in a doctor's office/hospital or at a testing site (if you did have symptoms) or have you or the child ever been tested for COVID (even if you did not have symptoms?)?	☐ Yes Child tested positive<br>☐ Yes Adult tested positive<br>☐ Yes, Child tested negative<br>☐ Yes, Both tested negative<br>☐ Yes adult tested negative<br>☐ No, neither was tested in a hospital/doctor's office
What were the dates that you or or your children were tested? (List multiple dates for multiple times)	______________________
Were you or your child hospitalized?	☐ Yes child<br>☐ Yes Parent<br>☐ No Neither
Were either you or your child in the intensive care unit?	☐ Yes child<br>☐ Yes adult<br>☐ No Neither
Has anyone else in your household had symptoms of COVID-19 (thinking back from March until now)?	☐ Yes other adult (relative)<br>☐ Yes other child (relative)<br>☐ Yes other adult (non-relative)<br>☐ Yes other child (non-relative)<br>☐ No

Have any of these symptoms been confirmed by testing at a hospital or clinic for COVID-19 (if they did have symptoms) or has anyone in your household ever been tested for COVID (whether they had symptoms or not?)?	◯ Yes confirmed positive<br>◯ Yes confirmed negative<br>◯ No, not tested
What were the dates that these other people were tested (you can list multiple dates)	______________________
How many other people?	______________________
What is the Relationship of this (these) persons to you?	_____________________
What were the symptoms that were relevant for you?	☐ Cough<br>☐ Fever<br>☐ Muscle aches<br>☐ Stomach Ache/Diarrhea/Vomiting<br>☐ Sore Throat<br>☐ Shortness of Breath<br>☐ COVID toes<br>☐ Loss of smell/test
What was the date that you had symptoms?	______________________
What were the symptoms that were present for your child in terms of covid-19?	☐ Cough<br>☐ Fever<br>☐ Muscle Aches<br>☐ Stomach ace/diarrhea/vomiting<br>☐ Sore throat<br>☐ Shortness of breath<br>☐ COVID Toes<br>☐ Loss of smell/taste
What were the other household member symptoms?	☐ Cough<br>☐ Fever<br>☐ Muscle aches<br>☐ Stomach ache/diarrhea/vomiting<br>☐ Sore throat<br>☐ Shortness of breath<br>☐ COVID Toes<br>☐ Loss of smell/taste
What was the date(s) that the other household member had symptoms?	______________________
How long did these people have symptoms (in days?)	______________________
What date did your child have any of these symptoms?	______________________
How many people live in your house?	____________

How many bedrooms in your house?	__________________________
How many bathrooms in your house?	________ _______________
How many siblings does your child have that live in the house or how many other children live in the house?	___________________________
How many people usually eat breakfast or dinner together?	____________________________
How many people in your house share a bathroom on average?	__________________________
How many people in your house share a bedroom on average?	
How many caregivers does your child regularly have now during the shelter in place orders (adults - can be parents)?	________________1___________
Since the lockdown/shelter in place, does your child regularly play in the park or the street?	◯ Yes<br>◯ No
If so, how many days a week?	____________________________
Do you regularly go shopping in the lockdown?	◯ Yes<br>◯ No
If so (you go shopping), how many times a week do you go shopping?	_
Does your child regularly go with your shopping or to the store?	◯ Yes<br>◯ No
If your child goes with you to the store, how many times a week does this usually happen?	____________________________
Does your child regularly attend a daycare or go to a babysitter's house?	◯ Yes<br>◯ No
If your child sees a babysitter, goes to a daycare, how many times a week is this?	____ ____________________
Do you or your spouse/partner currently have employment or a job?	◯ Yes both<br>◯ Yes me<br>◯ Yes he/she does<br>◯ No Neither
What is your current employment?	___ _______

What is your spouse's/partner's employment?	__________ ______________
What is your profession?	___________
What is your spouse's profession?	__________________________
Can you confirm your current address?	
How many days a week have you been working on average during the shelter in place?	_
How many days a week has your partner/spouse been working during the shelter in place/lockdown?	________ _____________________
When you go out of the house do you regularly wear masks?	◯ Yes always<br>◯ Yes sometimes<br>◯ Yes occasionally<br>◯ No never
When you leave the house do you take public transportation (since the lockdown)?	◯ Yes always<br>◯ Yes sometimes<br>◯ Yes occasionally<br>◯ No never
